# Enhanced Activity and Stability of Heteroatom-Doped Carbon/Bimetal Oxide for Efficient Water-Splitting Reaction

**DOI:** 10.3390/polym15173588

**Published:** 2023-08-29

**Authors:** Thirukumaran Periyasamy, Shakila Parveen Asrafali, Ayoung Jang, Seong-Cheol Kim, Jaewoong Lee

**Affiliations:** 1 Department of Fiber System Engineering, Yeungnam University, Gyeongsan 38541, Republic of Korea; thirukumaran@ynu.ac.kr (T.P.); jayo1223@naver.com (A.J.); 2 School of Chemical Engineering, Yeungnam University, Gyeongsan 38541, Republic of Koreasckim07@ynu.ac.kr (S.-C.K.)

**Keywords:** hetero atom, porous carbon, calcination, electrocatalyst, water splitting

## Abstract

The research community is actively exploring ways to create cost-efficient and high-performing electrocatalysts for the oxygen evolution reaction. In this investigation, an innovative technique was employed to produce heteroatom-doped carbon containing NiCo oxides, i.e., HC/NiCo oxide@800, in the form of a three-dimensional hierarchical flower. This method involved the reduction of a bimetallic (Ni, Co) metal–organic framework, followed by carefully controlled oxidative calcination. The resulting porous flower-like structure possess numerous advantages, such as expansive specific surface areas, excellent conductivity, and multiple electrocatalytic active sites for both hydrogen and oxygen evolution reactions. Moreover, the presence of oxygen vacancies within HC/NiCo oxide@800 significantly enhances the conductivity of the NiCo substance, thus expediting the kinetics of both the processes. These benefits work together synergistically to enhance the electrocatalytic performance of HC/NiCo oxide@800. Empirical findings reveal that HC/NiCo oxide@800 electrocatalysts demonstrate exceptional catalytic activity, minimal overpotential, and remarkable stability when deployed for both hydrogen evolution and oxygen evolution reactions in alkaline environments. This investigation introduces a fresh avenue for creating porous composite electrocatalysts by transforming metal–organic frameworks with controllable structures. This approach holds promise for advancing electrochemical energy conversion devices by facilitating the development of efficient and customizable electrocatalytic materials.

## 1. Introduction

A sustainable way to produce hydrogen is water electrolysis. Until now, only 4% of H_2_ has been produced through water splitting [1,2,3]. The overall electrochemical water splitting includes hydrogen evolution at the cathode and oxygen evolution at the anode, which is represented in the following equations [1,2,3,4,5,6]:

In acidic solution

(1) $$\mathrm{At}\;\mathrm{cathode}:\;2H^{+}+2e^{-}\to H_{2}$$

(2) $$\mathrm{At}\;\mathrm{anode}:\;H_{2}O\to 2H^{+}+\frac{1}{2}O_{2}$$

(3) $$\mathrm{Overall}\;\mathrm{reaction}:\;H_{2}O\to H_{2}+\frac{1}{2}O_{2}$$

In neutral and alkaline solution

(4) $$\mathrm{At}\;\mathrm{cathode}:\;2H_{2}O+2e^{-}\to H_{2}+2{OH}^{-}$$

(5) $$\mathrm{At}\;\mathrm{anode}:\;2{OH}^{-}\to H_{2}O+\frac{1}{2}O_{2}+2e^{-}$$

(6) $$\mathrm{Overall}\;\mathrm{reaction}:\;H_{2}O\to H_{2}+\frac{1}{2}O_{2}$$

The kinetics of the overall reaction depend on an oxygen evolution reaction (OER), as OER is a kinetically sluggish process [4,5,6,7,8]. Therefore, an efficient electrocatalyst to overcome the sluggish kinetics of OER and thus reduce the activation energy barrier of both the OER and HER (hydrogen evolution reaction) is required. In this regard, commercial electrocatalysts based on noble metal (Pt) and metal oxides (RuO_2_/IrO_2_) are available, in terms of which Pt is effective regarding HER and RuO_2_/IrO_2_ is effective regarding OER [8,9,10,11]. But due to their high cost and low availability, their large-scale production is hindered. Therefore, there is an urge to synthesize non-precious electrocatalysts. With innumerable research works, it was found that non-noble metals, such as transition metal alloys, oxides, hydroxides, (oxy) hydroxides, carbides, phosphides, and chalcogenides were found to perform as effective electrocatalysts regarding HER and OER, and moreover, these materials are cheap and are abundantly available [12,13,14,15,16,17,18]. Among the above mentioned materials, transition metal oxides, in particular, bimetal oxides (where ‘A’ and ‘B’ represent transition metals) are preferred due to their variable valence state, tunable morphology, and enhanced redox stability. The bimetal oxide formed from Ni and Co was found to be efficient regarding HER and OER performances due to their synergistic effect produced by multiple valence states and their resistance regarding alkaline electrolyte [19,20,21,22,23].

Metal oxides along with carbon materials can perform as a proper electrocatalyst, as carbon materials form a base structure and support the metal oxide growth on it with suitable morphology [24,25]. Instead of using pure carbon material, heteroatom-doped carbon (HC) materials like nitrogen-, oxygen-, phosphorous- and sulfur-doped carbon materials are effective, as these dopants (i) enhance the catalytic performance by increasing the interfacial charge transfer between the bimetal oxides and HC, and (ii) improve the stability of the electrocatalysts by binding well with the bimetallic species. The catalytic activity of these hybrid electrocatalysts (bimetal oxides along with HC) depends on their morphology, structure, dimension, crystal structure orientation, and the number of active sites. Therefore, an appropriate synthetic strategy should be adopted to extract the electrocatalytic property of the synthesized materials to their fullest [26,27,28,29,30].

There are many reports related to Ni- and Co-based electrocatalysts that could efficiently enhance the water-splitting process. Martini et al. [31]. synthesized nanocomposites with Ni and Co salts along with MoSe_2_ and MoS_2_ through hydrothermal and calcination methods. The work revealed that the synthesized nanocomposites, i.e., NiCoMo, CoMoSeS, CoMo, and NiCoMoSe, act as effective electrocatalysts regarding OER, with an overpotential at 10 mA cm^−2^ current density of 356, 375, 375, and 390 mV, respectively. Wen et al. [32] synthesized two different electrochemically active materials, viz., CoNi-ZIF-67 and hybrid CoNi-ZIF-67@Ti_3_C_2_Tx, through co-precipitation reaction. The work reports that the hybrid material, CoNi-ZIF-67@Ti_3_C_2_T_x_, is effective regarding OER, showing a lower overpotential of 275 mV at a current density of 10 mA cm^−2^ than the pure CoNi-ZIF-67 (overpotential = 341 mV at η_j_ = 10 mA cm^−2^). Zhang et al. [33] synthesized two different electrocatalysts, CoNi-MOFs and CoNi-MOFs-DBD, with multiple active sites by creating a metal–organic framework (MOF) and adopting low-temperature dielectric barrier discharge plasma (DBD). The electrocatalysts, CoNi-MOFs-DBD, show a lower overpotential of 563 and 168 mV, respectively, at a current density of 40 mA cm^−2^ regarding OER and overpotential of 322 and 203 mV, respectively, at a current density of 10 mA cm^−2^, regarding HER.

In all these works, the authors modified the metallic counterpart by preparing alloys, oxides, hydroxides, sulfurization, selenization, and creating oxygen vacancies, but the carbon part is not given much importance. Hence, in this work, we emphasized on both metal and carbon parts. In case of metal, we preferred the bimetallic oxides of Ni and Co to create multiple vacancies. The carbon part, containing different heteroatoms, was prepared from a polymer source (polybenzoxazine). The combination of heteroatom-containing carbon with bimetallic oxides can result in a 3D hierarchical structure when hydrothermal and calcination methods are adopted. The performance of the electrocatalyst regarding OER and HER was examined and discussed in detail.

## 2. Materials

Eugenol (98%), and paraformaldehyde (95%) were purchased from Sigma-Aldrich (St. Louis, MO, USA). Ethylene diamine (99%), potassium hydroxide (KOH) (85%), sodium hydroxide (NaOH) (97%) and dimethyl sulfoxide (DMSO) (99.9%) were purchased from Duksan Chemicals Co., Ltd., Suji-gu Yongin, Republic of Korea. Nickel nitrate hexahydrate, cobalt nitrate tetrahydrate, polyvinylidene fluoride (PVDF) (99%) and N, N-dimethylformamide (DMF) (99.8%) were purchased from Duksan Chemicals Co., Ltd., Republic of Korea. All chemicals were used without further purification.

## 3. Methods

### 3.1. Synthesis of Hetero-Doped Carbon from Benzoxazine

The production of hetero-doped carbon from benzoxazine involves a multi-step procedure commencing with the synthesis of the benzoxazine monomer, referred to as Eu-Bzo. Eu-Bzo is derived through Mannich condensation, where ethylenediamine (3 g), eugenol (16.42 g), and paraformaldehyde (6 g) were taken in 1:2:4 molar ratio and reacted for 3 h at 120 °C. The resulting product, containing Eu-Bzo, is precipitated in NaOH solution and left to dry completely overnight. Subsequently, the monomer undergoes a step-wise curing process up to 250 °C, transforming into a self-curing thermoset polymer known as poly(Eu-Bzo). The hetero-doped carbon was synthesized from this polymer. The formed polymer is subjected to heating at 600 °C in a nitrogen atmosphere, effectively eliminating oxygen and leaving behind a carbon skeleton. Next, the carbonized material undergoes activation with aqueous KOH (the ratio of KOH and carbon material is 2:1). This activation process introduces heteroatoms, such as nitrogen and oxygen, into the carbon skeleton. Further activation of the resulting material occurs in a tubular furnace at 600 °C, leading to the production of the final product, HC (hetero-doped carbon).

### 3.2. Synthesis of HC/NiCo Oxides

**Scheme 1:**
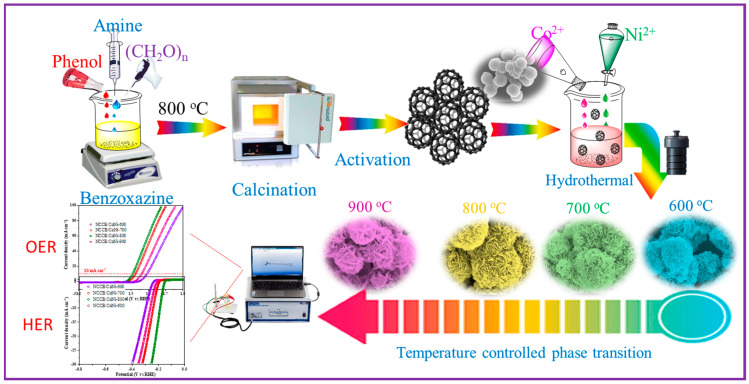
Schematic diagram of the preparation of HC/NiCo oxides at different temperature.

The synthesis of HC/NiCo oxide@600 begins with the dissolution of 10 mg of HC, Ni(NO_3_)_2_·6H_2_O, and Co(NO_3_)_2_·4H_2_O in a mixed solvent of H_2_O/DMF/EtOH (1:1:1, *v*/*v*) with a 2:1 mole ratio. The solution is stirred at room temperature for 60 min. This step allows the metal salts to dissolve and interact with each other. Afterward, the resulting suspension is aged at room temperature for 12 h while air is bubbled throughout the process. This step oxidizes Ni and Co, which is necessary for the formation of the HC/NiCo oxide@600 composite. Next, the solution is sealed in a 100 mL Teflon-lined stainless-steel autoclave and heated at 120 °C for 24 h. This step causes the metal salts to react and form the HC/NiCo oxide@600 composite. Once cooled to room temperature, the black powder formed is washed several times with DMF and EtOH and then dried overnight at 100 °C. This step removes any residual solvent and metal salts from the composite. For HC/NiCo oxide@600, the as-synthesized HC/NiCo (30 mg) is transferred to a tube furnace and heated up to 600 °C at a rate of 5 °C min^−1^ under a nitrogen atmosphere for 2 h. This step further stabilizes the composite and gives it its final properties. Sample-700, Sample-800, and Sample-900 were prepared using the same procedure, but with different calcination temperatures of 700, 800, and 900 °C, respectively (Scheme 1).

### 3.3. Fabrication of Working Electrode

The working electrodes were fabricated using four different materials: HC/NiCo oxide@600, HC/NiCo oxide@700, HC/NiCo oxide@800, and HC/NiCo oxide@900. The first step was to grind HC/NiCo oxide@600 and PVDF in a 95:5 weight ratio with N-methyl-2-pyrrolidone to create a homogeneous paste. This paste was then drop-casted onto Ni foam with a 1 cm^2^ surface area and dried for 48 h at 100 °C in a hot air oven. The other electrodes were prepared in a similar manner. Once the working electrodes were fabricated, they were evaluated for their electro-catalytic activity using a standard three-electrode system with 1 M KOH as the electrolyte. The electrochemical workstation CS2350 was used with Hg/HgO electrode and Pt foil as the reference and counter electrodes, respectively. The system was purged with high-purity O_2_ before the OER assessment, and the bubbling was maintained during the electrochemical experiment. All potentials were calibrated against the reversible hydrogen electrode (RHE) using the equation E_RHE_ = E_Hg/HgO_ + 0.0592 pH + 0.098 V. The electrochemical workstation automatically compensated for 90% of the iRs drop in each polarization curve. Various tests were performed, including cyclic voltammetry (CV) measurements at different scanning rates, linear sweep voltammetry (LSV) at a constant rate of 5 mV s^−1^, and Tafel slope calculations from the linear part of overpotential versus current density in log scale (log |j|).

## 4. Instrumentation

The prepared materials, i.e., HC/NiCo-600, HC/NiCo-700, HC/NiCo-800, and HC/NiCo-900 were characterized by various physicochemical techniques such as field emission scanning electron microscopy (FESEM, Urbana, IL, USA) with energy-dispersive X-ray spectroscopy (EDS), high-resolution transmittance electron microscopy (HRTEM, Tokyo, Japan), X-ray diffraction (XRD), Raman spectroscopy, nitrogen adsorption–desorption isotherms and X-ray photoelectron spectroscopy (XPS). FESEM was carried out on a Hitachi S-4800 (Tokoy, Japan) equipped with EDX at an accelerating voltage of 4 kV. TEM/HRTEM images were performed with an FEI-Tecnai TF-20 transmission electron microscope with an operating accelerating voltage of 120 kV (Hillsboro, OR, USA). XRD measurements were carried out using a PANalytical X’Pert3 MRD diffractometer with monochromatized Cu Kα radiation (λ = 1.54 Å) at 40 kV and 30 mA and were recorded in the range from 10 to 80° (2θ) (Malvern, UK). The Raman spectrum was recorded on an XploRA Micro-Raman spectrophotometer (Horiba, Palaiseau, France) with a range of 500 to 4000 cm^−1^. Nitrogen sorption isotherms were measured at −197 °C using a Micromeritics ASAP 2000. Before the experiments, the samples were dried at 120 °C and evacuated for 8 h in flowing argon at the flow rate of 60 standard cubic centimeters per minute at 140 °C. Surface area, pore size, and pore volumes were obtained from isotherms using the conventional Brunauer–Emmet–Teller (BET Raymond Ave., Fullerton, CA, USA) and Barrett–Joyner–Halenda (BJH) equations. XPS spectra were achieved using a K-Alpha (Thermo Scientific, Waltham, MA, USA). CasaXPS software version number (2.3.22PR1.0) was used for the deconvolution of the high-resolution XPS spectra. All the instrumentation analyses were undertaken at the core research support center for natural products and medical materials of Yeungnam University.

## 5. Results and Discussion

The 3D hierarchical flower-like structure of HC/NiCo oxide@800 was synthesized through a two-step process. Initially, a hydrothermal reaction at 120 °C for 24 h introduced bimetallic components into the carbon framework. Subsequently, the HC/NiCo material underwent calcination at various temperatures ranging from 600 to 900 °C. It was observed that the optimal temperature for obtaining the desired 3D structure was 800 °C. Thus, the calcination temperature significantly influences the formation of the 3D hierarchical morphology. To gain further insights into the structure and composition, the HC/NiCo oxide samples formed at different calcination temperatures (600, 700, 800, and 900 °C) were subjected to preliminary analysis.

### 5.1. XRD

**Figure 1:**
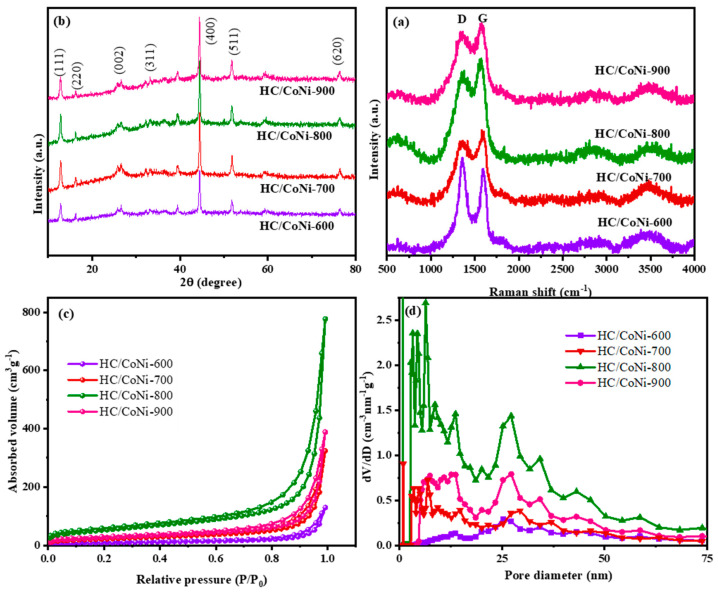
(**a**) XRD; (**b**) Raman; (**c**) BET; and (**d**) pore size distribution of HC/NiCo oxide at different calcination temperatures.

X-ray diffraction (XRD) analysis was used to investigate the phase purity and crystallinity of HC/NiCo oxide at various calcination temperatures. The XRD patterns of HC/NiCo, calcined at different temperatures, are shown in Figure 1a. The figure reveals that the diffraction peaks correspond to the cubic planes of NiCo oxide [(111), (220), (311), (400), (511), and (620)] at distinct 2*θ* values. These peaks indicate that the NiCo oxides are well crystallized and have a high degree of phase purity (JCPDS no. 73-1702). In addition to the NiCo peaks, a broad diffraction peak is observed between 15 and 30°. This peak is attributed to the characteristic (002) plane of the hexagonal graphitic structure of carbon. The broadening of this peak suggests that the carbon in HC/NiCo oxide is not highly graphitized [34,35,36,37]. This is likely due to the presence of the bimetallic NiCo oxide, which disrupts the ordered arrangement of the carbon atoms. As a result, the carbon in HC/NiCo oxide has a lower degree of graphitization than pure carbon [11,14,19].

The results of the XRD analysis show that HC/NiCo oxide is a highly crystalline material with a high degree of phase purity. However, the carbon in HC/NiCo oxide is not highly graphitized, likely due to the presence of the bimetallic NiCo oxide. This has implications for the catalytic properties of HC/NiCo oxide, as the degree of graphitization of the carbon can affect the activity and selectivity of the catalyst. In addition to the XRD analysis, other techniques can be used to investigate the structure and properties of HC/NiCo oxide. For example, SEM and TEM can be used to visualize the morphology of the NiCo oxide and the carbon matrix. Raman spectroscopy can be used to determine the degree of graphitization of the carbon. These techniques can provide further insights into the structure and properties of HC/NiCo oxide, which can be used to design more efficient and selective catalysts.

### 5.2. Raman

Figure 1b illustrates the Raman spectra of HC/NiCo oxide at various calcination temperatures. The characteristic D-band and G-band of carbon materials appear at 1336 and 1575 cm^−1^, respectively. The degree of graphitization of carbon materials is assessed using the R value, which is obtained by the I_D_/I_G_ peak ratio. The calculated peak intensities between the D and G bands for HC/NiCo oxide@600, HC/NiCo oxide@700, HC/NiCo oxide@800, and HC/NiCo oxide@900 are 1.12, 0.93, 0.93, and 0.96, respectively. This R value indicates an increase in structural defects in the carbon material due to the inclusion of bimetallic oxides. Furthermore, all spectra exhibit a broad peak at 619 cm^−1^, indicating the formation of metal–oxygen–metal bonds [29,38,39,40,41,42]. The intensity of this peak is notably more pronounced and broader in HC/NiCo oxide@800 compared to the others, indicating that 800 °C is the optimal temperature for bimetallic oxide formation within the carbon interstices.

### 5.3. BET Analysis

The porosity characteristics of the materials under investigation were analyzed through BET analysis. Figure 1c,d present the N_2_ adsorption/desorption isotherms and pore size distribution (PSD) for these materials. All prepared samples exhibited a typical type IV isotherm, indicating the presence of mesopores within their structure. The overlapping adsorption and desorption isotherms suggested the co-existence of micropores and mesopores in the material’s framework. The PSD analysis revealed that the majority of pores for all samples fall within 10–50 nm in diameter. Particularly, HC/NiCo oxide@800 displayed a significant number of pores with diameters between 3–12 nm and 26 nm. The material synthesized at 800 °C demonstrated a well-distributed porous structure containing both micropores and mesopores [42,43]. The BET surface area measurements for HC/NiCo oxide@600, HC/NiCo oxide@700, HC/NiCo oxide@800, and HC/NiCo oxide@900 were 224, 235, 286, and 240 m^2^ g^−1^, respectively. These results indicate that a calcination temperature of 800 °C yielded an optimal hierarchical porous structure with a suitable surface area. This favorable structure enables efficient ion adsorption and desorption at an accelerated rate while maintaining robust mechanical integrity.

### 5.4. XPS Analysis

**Figure 2:**
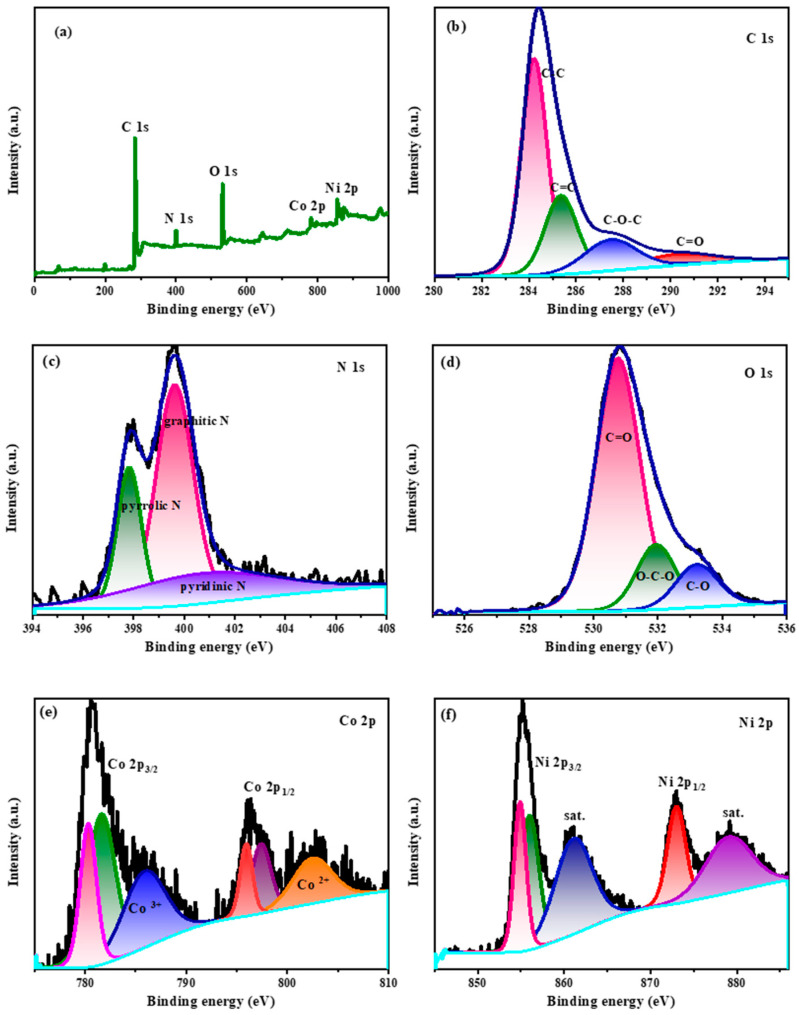
XPS spectra of HC/NiCo oxide@800 showing (**a**) survey spectrum; and (**b**–**f**) deconvoluted spectrum of each components.

Figure 2 presents the results of an XPS analysis of the surface chemical composition and valence state of HC/NiCo oxide@800. The survey spectrum (Figure 2a) confirms the presence of C, N, O, Co, and Ni elements. The intensity of the O 1s peak is greater than that of the N 1s peak, suggesting that the oxygen atoms come from both the carbon surface and the formation of bimetallic oxides. To gain a deeper understanding of the surface chemistry, the spectra for each element were deconvoluted. The high-resolution C 1s spectrum (Figure 2b) is resolved into four peaks: 284.2 eV (C=C carbon of the aromatic ring), 285.4 eV (C–C single bond), 287.5 eV (carbonyl carbon, C=O/C–O), and 290.6 eV (C–N carbon). The N 1s spectrum (Figure 2c) is deconvoluted into three peaks, representing different nitrogen forms: pyridinic N at 397.8 eV, pyrrolic N at 399.6 eV, and quaternary N at 401.3 eV. The presence of these different nitrogen species enhances the capacitance of the material, with pyridinic and pyrrolic N participating in pseudo-capacitive interactions and quaternary N improving the carbon material’s conductivity through efficient ion transfer. The O 1s spectrum (Figure 2d) is deconvoluted into three peaks: a dominant peak at 530.7 eV attributed to metal–oxygen bonds, and two smaller peaks at 531.9 and 533.1 eV corresponding to surface-adsorbed hydroxyl (–OH) and carbonyl (C–O) oxygens, respectively. The presence of oxygen atoms enhances the wettability of the electrode material and its overall capacitance. The bimetallic components, Co and Ni, were also analyzed. The deconvoluted spectrum of Co 2p (Figure 2e) shows two spin states at 781.6 and 797.4 eV corresponding to Co 2p_3/2_ and Co 2p_1/2_, respectively, along with satellite peaks indicating the coexistence of Co^2+^ and Co^3+^. Similarly, the deconvoluted spectrum of Ni 2p (Figure 2f) exhibits two spin-orbit doublets at 854.8 and 873.1 eV, representing Ni 2p_3/2_ and Ni 2p_1/2_ spin states, and two satellite bands indicating the presence of Ni^2+^ state. The incorporation of bimetallic oxides into the carbon framework facilitates rapid redox reactions, thereby enhancing pseudo-capacitance. Overall, the XPS analysis demonstrates the successful integration of bimetallic oxides into the carbon structure. This offers intriguing prospects for improved performance in energy-storage applications [9,26,35].

### 5.5. SEM Analysis

**Figure 3:**
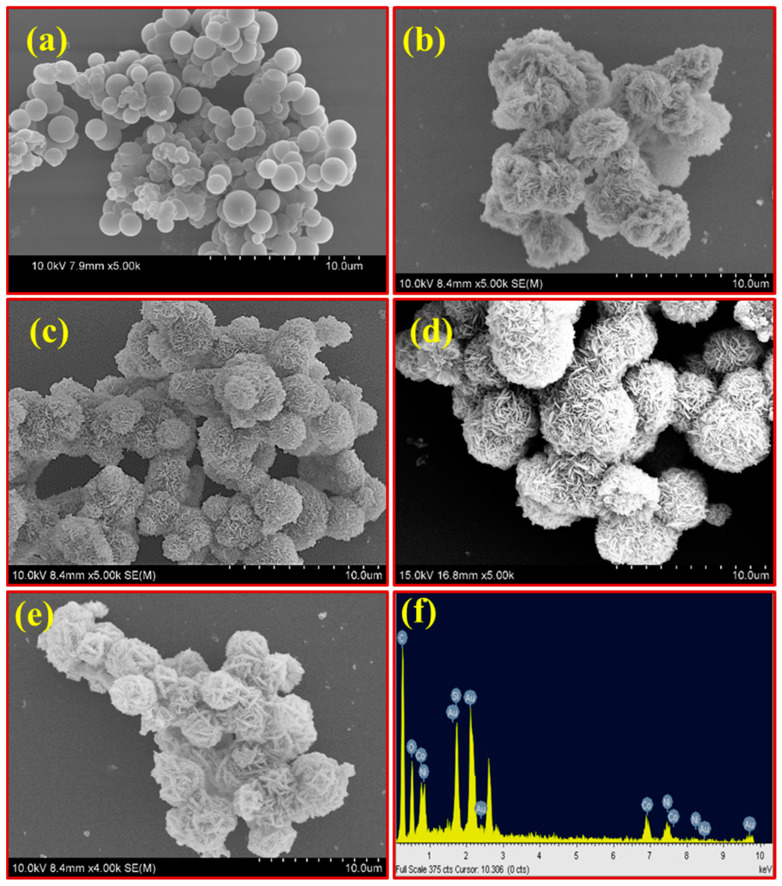
SEM images of (**a**) neat HC; (**b**) HC/NiCo oxide@600; (**c**) HC/NiCo oxide@700; (**d**) HC/NiCo oxide@800; (**e**) HC/NiCo oxide@900; and (**f**) EDX spectrum of HC/NiCo oxide@800.

Field-emission scanning electron microscopy (FESEM) was employed to analyze the morphology of HC/NiCo oxide samples synthesized at different calcination temperatures. Figure 3a–f illustrates SEM images of pristine carbon, HC/NiCo oxide at various calcination temperatures, and the EDX spectrum of HC/NiCo oxide@800. The SEM image of pristine carbon (Figure 3a) reveals a smooth, spherical shape, exhibiting multiple interconnected particles of different sizes. However, after incorporating bimetallic oxides and subjecting them to calcination, a remarkable transformation from a spherical to a flower-like structure occurred. The flower-like morphology emerged through a process wherein the carbon material laid the foundation in the form of a basic spherical structure, on which the bimetallic oxides flourished, giving rise to a 2D petal-like formation that ultimately culminated in an intricate 3D hierarchical flower-like architecture. Clearly, the calcination temperature played a pivotal role in this transformation, as depicted in the SEM images. At 600 and 700 °C (Figure 3b,c), the calcination temperature was insufficient to achieve a complete 3D structure. Consequently, the flowers were inadequately formed, with noticeable instances of broken petals. At 800 °C (Figure 3d), the optimal calcination temperature, a fully developed 3D hierarchical flower with closely aligned petals manifested. On the other hand, when the temperature was raised to 900 °C (Figure 3e), the petals clustered together, resulting in a diminished flower morphology with abundant void spaces. Moreover, some flower structures were damaged, yielding non-uniform shapes. These structural observations solidify the superiority of 800 °C as the ideal calcination temperature for obtaining a flower-like morphology. The intricate porous network structure, consisting of interlinked spheres with vertically aligned 2D petals, facilitates seamless charge transport and ion diffusion. The EDX spectrum of HC/NiCo oxide@800 (Figure 3f) further corroborates the presence of all elements (C, N, O, Co, and Ni) in their respective proportions.

### 5.6. TEM Analysis

**Figure 4:**
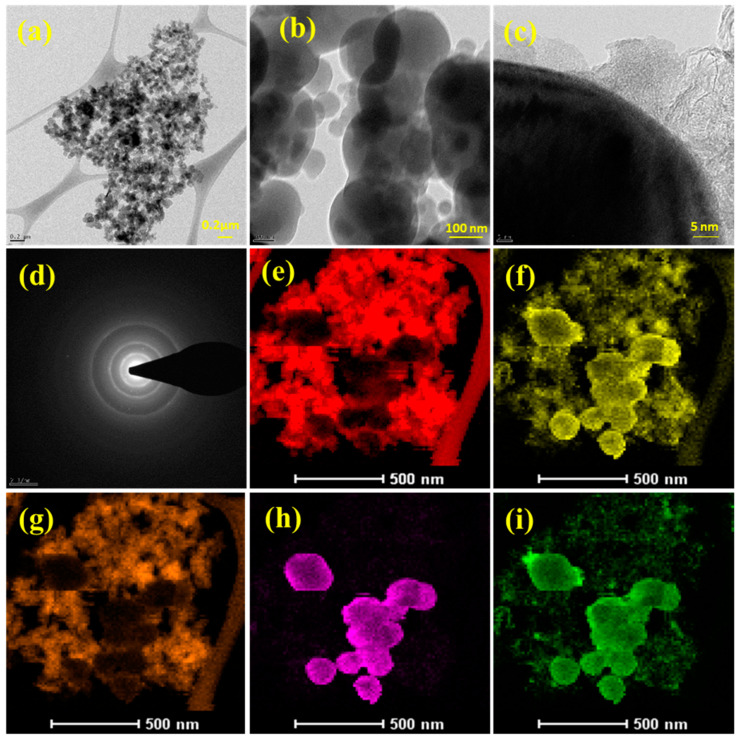
TEM images of HC/NiCo oxide@800 (**a**–**c**) at different magnifications; (**d**) SAED pattern; and (**e**–**i**) EDS mapping of all elements.

A comprehensive examination of the surface integration of HC/NiCo oxide@800 was conducted using high-resolution transmission electron microscopy (HRTEM). The results, presented in Figure 4a–i, encompass HRTEM images, selected area electron diffraction (SAED) patterns, and energy-dispersive X-ray spectroscopy (EDS) maps of HC/NiCo oxide@800. The TEM image in Figure 4a clearly exhibits a hierarchical structure with two distinct morphologies. At its base, there is an ultra-thin porous structure, adorned uniformly with sharp spikes throughout. The porous structure is derived from the carbon’s spherical shape, while the spike-like structure originates from the petal shape of the bimetallic oxides. This finding corroborates well with the SEM results. The TEM images at higher magnification (Figure 4b) reveals that the structure is densely packed without any signs of aggregation. This compact and well-anchored configuration, comprising conductive carbon material infused with bimetallic oxides, facilitates highly active interfaces and redox centers, thus enhancing supercapacitor performance. The SAED pattern (Figure 4c) distinctly displays the different phases of the bimetallic oxides. Moreover, the EDS maps confirm the uniform distribution of all elements, namely C, N, O, Co, and Ni, present in it. This substantiates that Co and Ni are uniformly embedded within the carbon framework (Figure 4d–i).The combination of these preliminary characterizations provides strong evidence that HC/NiCo oxide@800 has been successfully synthesized with the desired morphology, making it well-suited for use as an electrode material in electrochemical applications.

### 5.7. Electrochemical Studies

**Figure 5:**
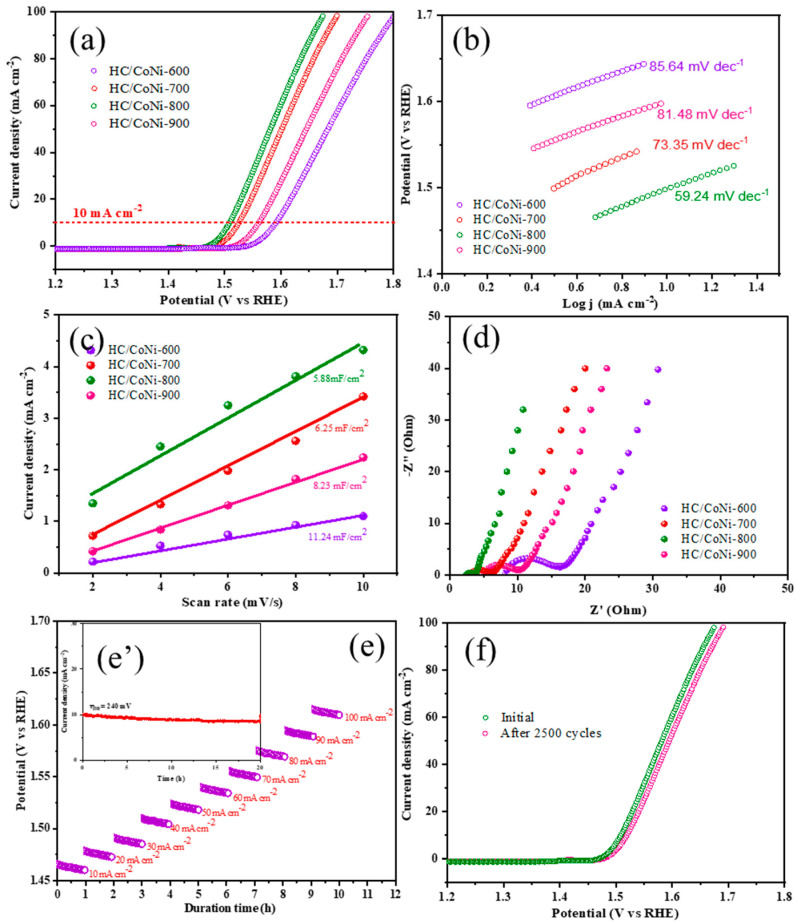
Electrochemical performance of various catalysts in 1 M KOH showing (**a**) polarization curves; (**b**) Tafel plots; (**c**) C_dl_ measurement at the non-Faradaic potential window; (**d**) Nyquist plots; (**e**) chronoamperometric plots at multiple current densities from 10 to 100 mA⋅cm^−2^ for HC/NiCo oxide@800; (**e′**) Inset: @ 10 mA cm^−2^; and (**f**) LSV curves before and after stability measurements.

The synergistic effect of bimetal oxides and heteroatom-doped carbon (HC/NiCo oxide) is expected to enhance the OER activity in water-splitting process. The electrochemical performance of the synthesized materials was analyzed using a three electrode system, where HC/NiCo oxide is used as working electrode against Hg/HgO as reference electrode with Pt as counter electrode using 1 M KOH electrolytic solution. Figure 5 depicts the electrochemical measurements of HC/NiCo oxide electrodes toward OER. As can be seen from the LSV measurements (Figure 5a), the overpotential corresponding to the current density of 10 mA/cm^−2^ was found to be 350 mV for HC/NiCo oxide@600; 295 mV for HC/NiCo oxide@700; 280 mV for HC/NiCo oxide@800; and 330 mV for HC/NiCo oxide@900. Among the prepared materials, HC/NiCo oxide@800 exhibits the lowest overpotential of 280 mV with the lowest Tafel slope of 59.24 mV dec^−1^ (Figure 5b). This could be attributed to the fact that the bimetallic oxides are enclosed by the carbon material, which prevents the corrosion of bimetals in an alkali solution. By doing so, the carbon material paves the way for electron transition from the transition metal oxides and thus accelerates the oxygen evolution reaction. As can be evidenced by the SEM images, a proper morphology is obtained at a calcination temperature of 800 °C and thus HC/NiCo oxide@800 can provide a continuous pathway for electron transfer through the hierarchical 3D morphology, exhibiting a lower overpotential value. These overpotential values are much lower or comparable to previously reported works: 356 mV for NiCoMo [4]; 275 mV for CoNi-ZIF67 [5]; 563 mV at 40 mA cm^−2^ current density for CoNi-MOFs-DBD; and 290 mV for NC-2@CoO. Figure 5c displays the double-layer capacitance obtained from the CV measurements in the non-Faradaic region at different scan rates. The C_dl_ values were found to be 11.24, 6.25, 5.88, and 8.23 mF cm^−2^ for HC/NiCo oxide@600, HC/NiCo oxide@700, HC/NiCo oxide@800, and HC/NiCo oxide@900, respectively. The higher C_dl_ value implies larger electrochemical surface area (ECSA). Even though HC/NiCo oxide@600 possess larger surface area, its surface is not effective in transferring electrons during the electrochemical process. Even with low surface area and proper morphology, HC/NiCo oxide@800 could effectively transfer electrons, showing enhanced electrocatalytic activity regarding OER [44,45,46].

Electrochemical impedance spectroscopy (EIS) measurements were performed to evaluate the kinetics of the electrocatalytic process. Figure 5d represents the EIS spectra of the prepared materials. The EIS spectra reveals that among the four materials, HC/NiCo oxide@800 has the lowest R_ct_ (charge transfer resistance) of 1.08 Ω, evidenced by the smallest semicircle, when compared with others (7.67 Ω for HC/NiCo oxide@600; 3.1 Ω for HC/NiCo oxide@700; and 4.8 Ω for HC/NiCo oxide@900). This is attributed to the fact that the interconnected 3D flower-like morphology formed from the HC and bimetal oxides paves the way for effective electron transfer from the highly conductive HC to bimetal oxides, thereby reducing their charge transfer resistance. The electrochemical stability of HC/NiCo oxide@800 was measured at different potentials with varying current densities from 10 to 100 mA cm^−2^ for 12 h and displayed in Figure 5e, and the inset of the figure displays the stability at 10 mA cm^−2^ current density for 20 h. There is only a very slight decrease in current densities from lower to higher region, indicating the enhanced electrochemical stability of the prepared material. Figure 5f depicts the LSV curves before and after the stability measurement, showing a slight shift from the original spectrum, indicating the long-term stability of the electrocatalytic material.

**Figure 6:**
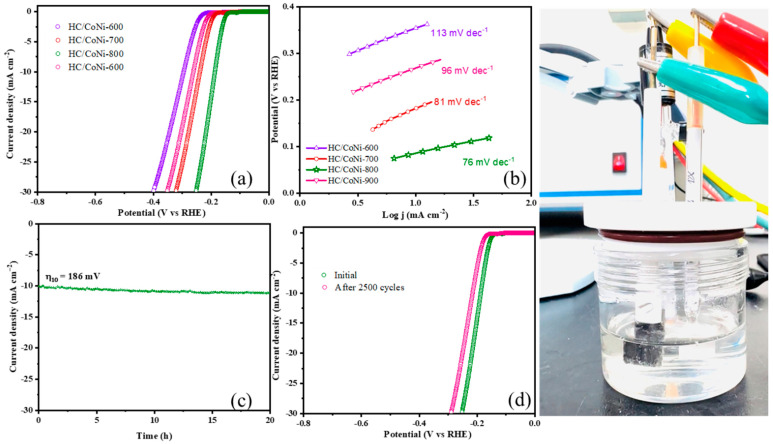
Electrochemical measurements regarding HER showing (**a**) polarization curves; (**b**) Tafel plots; (**c**) electrochemical stability at 10 mA cm^−2^ current density for 20 h; and (**d**) LSV curves before and after stability measurements.

The electrocatalytic behavior of the prepared materials regarding HER was measured using LSV and depicted in Figure 6a. The figure shows an overpotential value at a current density of 10 mA cm^−2^ to be 300, 250, 186 and 275 mV for HC/NiCo oxide@600, HC/NiCo oxide@700, HC/NiCo oxide@800, and HC/NiCo oxide@900, respectively. Tafel plots were constructed using logarithmic current density against potential, represented in Figure 6b, and the Tafel slopes were found to be 113 mV dec^−1^ for HC/NiCo oxide@600; 81 mV dec^−1^ for HC/NiCo oxide@700; 76 mV dec^−1^ for HC/NiCo oxide@800; and 96 mV dec^−1^ for HC/NiCo oxide@900. It could be seen that HC/NiCo oxide@800 has the lowest overpotential value of 186 mV and a Tafel slope value of 76 mV dec^−1^. This proves that a perfect 3D hierarchical structure along with sufficient surface area helps in creating more active sites that could predominantly transfer electrons continuously via interconnected petal-like morphology and thus result in lower overpotential value. The obtained value regarding HER is much lower than the previously reported works; using Co and Ni: at a current density of 10 mA cm^−2^, CoNi-MOFs displayed an overpotential of 322 mV, and CoNi-MOFs-DBD-2mins displayed an overpotential of 203 mV regarding HER [47,48]. It can be clearly visualized that bimetallic oxides along with heteroatom-doped porous carbon synergistically improved the electrocatalytic performance. Moreover, the electrochemical stability of HC/NiCo oxide@800 was assessed at a current density of 10 mA cm^−2^, represented in Figure 6c, exhibiting excellent stability for 20 h. The LSV (Figure 6d), measured after 20 h, shows a very slight variation from the LSV measurement taken before stability analysis. This further proves that HC/NiCo oxide@800 has excellent structural stability that could be retained even after stability measurements.

## 6. Conclusions

An efficient bifunctional electrocatalyst, HC/NiCo oxide@800, for water splitting, was prepared successfully by adopting simple and effective hydrothermal and calcination methods. The synergistic effect of heteroatom-doped carbon and bimetallic oxides produces a stable three-dimensional hierarchical flower-like morphology at a calcination temperature of 800 °C. This morphology effectively transfers electrons from one place to another, thereby facilitating both the HER and OER processes involved in the water-splitting reaction. The carbon part produced from polybenzoxazine source is very useful as it contains both nitrogen and oxygen species along with the carbon material. As these hetero atoms are present in the ring structure of Pbz, the carbonaceous material derived from Pbz has a stable network of hetero atoms. The prepared electrocatalyst, HC/NiCo oxide@800, has a lowest overpotential and a Tafel slope of 280 mV and 59.24 mV dec^−1^ regarding OER, and 186 mV and 76 mV dec^−1^ regarding HER. The prepared electrocatalyst has a bifunctional role. The present work proves that along with modifying the metallic counterpart, modification of the carbon counterpart will be effective to produce a perfect electrocatalyst for hydrogen production from water splitting. This kind of material fabrication with proper morphology and porosity can be utilized for other energy-related applications, including supercapacitors and sensors.

## Data Availability

Not applicable.
